# Effects of Exercise on Cancer-Related Fatigue in Breast Cancer Patients: A Systematic Review and Meta-Analysis of Randomized Controlled Trials

**DOI:** 10.3390/life14081011

**Published:** 2024-08-14

**Authors:** Runyu Zhou, Zhuying Chen, Shiyan Zhang, Yushu Wang, Chiyang Zhang, Yuanyuan Lv, Laikang Yu

**Affiliations:** 1 Beijing Key Laboratory of Sports Performance and Skill Assessment, Beijing Sport University, Beijing 100084, China; 18953366022@163.com; 2 Department of Strength and Conditioning Assessment and Monitoring, Beijing Sport University, Beijing 100084, China; zhuying20232120126@126.com (Z.C.); 17634977464@163.com (S.Z.); 3 China Institute of Sport and Health Science, Beijing Sport University, Beijing 100084, China; m18756902144@167.com (Y.W.); 13215580747@163.com (C.Z.)

**Keywords:** exercise, cancer-related fatigue, breast cancer, systematic review, meta-analysis

## Abstract

The primary objective of this study was to assess the influence of exercise interventions on cancer-related fatigue (CRF), specifically in breast cancer patients, with the ultimate goal of establishing an optimal exercise prescription for breast cancer patients. A comprehensive search was undertaken across multiple databases, including Embase, PubMed, Cochrane Library, Web of Science, and Scopus, covering data published up to 1 September 2023. A meta-analysis was conducted to calculate the standardized mean difference (SMD) along with its corresponding 95% confidence interval (CI), thereby quantifying the effectiveness of exercise in alleviating CRF in the breast cancer patient population. Twenty-six studies met the inclusion criteria. Aerobic exercise (SMD, −0.17, *p* = 0.02), resistance exercise (SMD, −0.37, *p* = 0.0009), and combined exercise (SMD, −0.53, *p* < 0.0001) significantly improved CRF in breast cancer patients. In addition, exercise intervention conducted ≥3 times per week (SMD, −0.47, *p* = 0.0001) for >60 min per session (SMD, −0.63, *p* < 0.0001) and ≥180 min per week (SMD, −0.79, *p* < 0.0001) had greater effects on improving CRF in breast cancer patients, especially middle-aged patients (SMD, −0.42, *p* < 0.0001). Exercise is an effective approach to improving CRF in breast cancer patients. When devising an exercise program, the primary consideration should be the incorporation of combined exercise as the principal intervention. This entails ensuring that participants engage in the program at least three times weekly, with each session lasting for more than 60 min. The ultimate aim is to achieve a total weekly exercise duration of 180 min by progressively increasing the frequency of exercise sessions.

## 1. Introduction

Cancer stands as one of the most pervasive health conditions globally, encompassing an astonishing array of over 200 identified types that have been linked to cause more than 60 dysfunctions [1]. Alarmingly, both the global incidence and mortality rates of cancer have been escalating steadily over the past few decades, portending a grim future where it is projected to emerge as the primary cause of mortality and the foremost impediment to extending human life expectancy in the 21st century [2,3].

Notably, breast cancer occupies a particularly ominous position, ranking as the most frequently occurring cancer among women and the leading contributor to cancer-related fatalities [3]. According to the American Cancer Society, the incidence of breast cancer has continued to increase, with an annual increase of 0.6–1% from 2015 to 2019 [4,5]. In recent years, with the development and application of effective anti-tumor therapies, the mortality rate and risk of postoperative recurrence in breast cancer patients have been significantly reduced [6,7,8]. However, as survival rates increase, more patients are facing a range of quality-of-life issues related to breast cancer treatment, such as cancer-related fatigue (CRF), premature menopause, cognitive dysfunction, depression, and anxiety. Previous studies have shown that the overall quality of life of young women with breast cancer is significantly reduced [9,10,11].

CRF is a prevalent symptom encountered in breast cancer patients, with a staggering 60% of them enduring moderate to severe fatigue even a year post-diagnosis [12,13], which can significantly affect patients’ quality of life [14]. As per the National Comprehensive Cancer Network, CRF is characterized as a distressing, relentless, and subjective experience of exhaustion or a combination of physical, emotional, and/or cognitive fatigue, which stems from either the cancer itself or from its treatment. Notably, this fatigue does not abate with rest or sleep and significantly hampers an individual’s ability to function normally [15,16]. There is a suggested correlation between CRF and various physiological factors, including pro-inflammatory cytokines, hypothalamic–pituitary–adrenal axis dysregulation, circadian rhythm desynchronization, and skeletal muscle atrophy, but the precise mechanisms causing CRF remain incompletely understood [16,17,18,19]. CRF affects the quality of life of cancer patients and their ability to reintegrate into normal daily life [20].

It was once believed that cancer patients should avoid physical activity and prioritize rest to facilitate cancer treatment and recovery. However, excessive physical inactivity may lead to a deterioration in fitness and physical functioning, thereby promoting the development of CRF [21,22]. Currently, studies show that various exercise modalities can reduce CRF in breast cancer patients. Notably, aerobic exercise combined with relaxation training has been proven effective in substantially alleviating CRF in breast cancer patients [23]. Courneya et al. [24] emphasized the efficacy of aerobic exercise in postmenopausal breast cancer patients. In addition, Milne et al. [25] further validated the positive impact of exercise on post-treatment fatigue and physical function. However, Pagola et al. [26] found no significant reduction in fatigue after 16 weeks of combined exercise intervention. Similarly, Ergun et al. [27] found no notable differences in fatigue scores between pre- and post-intervention groups, regardless of exercise supervision or type. Furthermore, Furmaniak et al. [21] revealed that exercise during adjuvant therapy for breast cancer did not yield a clear improvement in fatigue. This discrepancy underscores the uncertainty surrounding the optimal exercise regimen (type, frequency, and duration) for effectively mitigating CRF in breast cancer patients. 

Therefore, the present meta-analysis, which builds upon rigorous randomized controlled trials (RCTs), aims to assess the effects of exercise on CRF and establish a definitive exercise prescription tailored to the needs of breast cancer patients.

## 2. Materials and Methods

### 2.1. Design

This study adhered to the rigorous guidelines outlined in the Preferred Reporting Items for Systematic Evaluation and Meta-Analysis (PRISMA, 2020) [28], ensuring the highest standards of methodology and reporting. The protocol has been officially registered with PROSPERO under the identification number CRD42023457710.

### 2.2. Search Strategy

To gather an exhaustive collection of relevant RCTs, a comprehensive literature search was conducted across 5 prestigious databases: Embase, PubMed, Cochrane Library, Web of Science, and Scopus. The search was limited to studies published up until 1 September 2023 and utilized a combination of the following keywords and Medical Subject Headings (MESH) terms: exercise, cancer, and fatigue. To supplement the search, the reference lists of the identified studies were manually screened for any additional potentially eligible articles. The screening and selection process was independently undertaken by two researchers (R.Z. and Z.C.). In cases where a disagreement arose, a third reviewer (L.Y.) was involved in the discussion, fostering a collaborative approach until a consensus was reached.

### 2.3. Eligibility Criteria

The inclusion criteria for the study were as follows: (1) RCT design; (2) participants were breast cancer patients; (3) there were both an intervention group and a control group; and (4) outcomes were assessed using a specific fatigue scale.

The exclusion criteria were as follows: (1) non-English publications; (2) review articles; (3) conference articles; (4) outcome indicators that could not be converted into mean and standard deviation (SD); and (5) studies without a control group.

### 2.4. Data Extraction

The process of data extraction was conducted independently by two authors (R.Z. and Z.C.), with a focus on the following key elements: (1) the primary author’s surname and the year in which the study was published; (2) sample size, age, and tumor stage; (3) the type of intervention, intervention duration, frequency, and session duration; (4) the outcome metrics that captured the variation in CRF.

### 2.5. Methodological Quality Assessment

The assessment of the risk of bias was carried out independently by two authors (R.Z. and Z.C.), and any discrepancies were resolved through discussion. The assessment was conducted using the Cochrane Randomized Trials Risk of Bias Tool (RoB-2) [29], which scrutinizes six domains: randomization sequence generation, allocation concealment, blinding, incomplete outcome data, choice of outcome report, and other biases. Each domain was assigned a risk level of “low”, “high”, or “unclear” [30].

### 2.6. Statistical Analysis

Since fatigue was assessed using different questionnaires, the data analysis employed a random-effects model to derive a standardized mean difference (SMD) alongside a 95% confidence interval (CI). Heterogeneity was assessed using the I^2^ statistic, with values of 0%, 25%, 50%, and 75% interpreted as indicating no, low, moderate, and high heterogeneity, respectively [31]. In case of high heterogeneity (I^2^ > 50%), additional analytical steps were undertaken, including subgroup analysis, meta-regression, and sensitivity analysis, to provide deeper insights into the results. The publication bias of the included studies was visualized by funnel plots. 

During the subgroup analyses, we endeavored to classify the included studies according to various intervention characteristics: the type of exercise (aerobic, resistance, combined exercise), frequency (less than 3 times weekly, 3 or more times weekly), session duration (up to 60 min per session, over 60 min per session), weekly time (less than 180 min weekly, 180 min or more weekly), and participants’ age (middle-aged, 45 ≤ age < 60; elderly, age ≥ 60). We utilized RevMan.5 software to create forest plots, while for meta-regression, sensitivity analysis, and the generation of funnel plots, Stata 17 software was employed. Outcomes were considered statistically significant if the *p*-value was less than 0.05.

## 3. Results

### 3.1. Studies Selection

**Figure 1:**
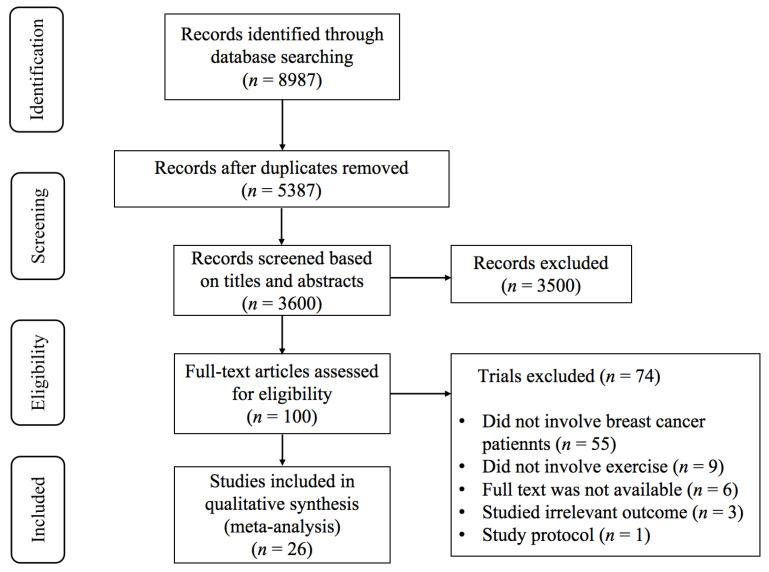
PRISMA flowchart of study selection.

As depicted in Figure 1, a comprehensive search across five databases yielded a total of 8987 pertinent studies. After eliminating duplicates, 3600 studies were assessed by reading titles and abstracts, resulting in the exclusion of 3500. Following a thorough evaluation of the full text, 74 studies were excluded due to the following reasons: (1) the studies did not involve breast cancer patients (*n* = 55); (2) the intervention did not involve exercise (*n* = 9); (3) the full text was not available (*n* = 6); (4) the investigated outcomes were irrelevant (*n* = 3); and (5) study protocol (*n* = 1). Finally, 26 studies [32,33,34,35,36,37,38,39,40,41,42,43,44,45,46,47,48,49,50,51,52,53,54,55,56,57] met the inclusion criteria.

### 3.2. Characteristics of the Included Studies

The key features of the interventions and participants are summarized in Table S1. The pooled studies encompassed 1258 patients in the intervention groups and 1049 patients in the control groups. Sample sizes ranged from 20 to 223 individuals across various studies. Eight studies utilized samples of over 100 breast cancer patients [37,40,42,43,51,54,56,57]. The age of the patients varied widely, with a mean range spanning from 45 to 66.6 years, and their breast cancer stages encompassed the entire spectrum, from stage 0 to stage 4. CRF was tested using the Functional Assessment of Chronic Illness Therapy-Fatigue (FACIT-F, five studies) [32,38,41,49,55]; the European Organization for Research and Treatment of Cancer Core Quality of Life Questionnaire-C30 (EORTC QLQ-C30, four studies) [33,36,42,47]; the Piper Fatigue Scale/the Revised Piper Fatigue Scale (PFS, five studies) [35,43,44,46,54]; the Multidimensional Fatigue Inventory (MFI/MFI-20, four studies) [40,42,56,57]; the Brief Fatigue Inventory (BFI, two studies) [39,52]; the Fatigue Quality List (FQL, two studies) [56,57]; the Profile of Mood States (POMS, one study) [34]; the Functional Assessment of Cancer Therapy-Anemia scale (FACT-An, one study) [37]; the Fatigue Severity Scale (FSS, one study) [45]; a 10 cm linear analog scale (one study) [48]; the Pittsburgh Fatigability Scale (PFS, one study) [50]; the Functional Assessment of Cancer Therapy-Endocrine Symptoms (FACT-ES, one study) [50]; the Fatigue Symptom Inventory (FSI, one study) [51]; and the Fatigue Assessment Questionnaire (FAQ, one study) [53]. Seven studies involved aerobic exercise [32,37,43,48,49,51,57], seven studies involved resistance exercise [36,37,41,44,46,52,53], and thirteen studies combined aerobic and resistance exercise [33,34,35,38,39,40,42,45,47,54,55,56,57]. The weekly intervention frequency varied, with some occurring as frequently as 5 times per week and as infrequently as once a week, averaging out to 3.2 times per week. The session duration ranged from a minimum of 15 min to a maximum of 90 min, with an average duration of 51.5 min per session. Lastly, the weekly time ranged from 40 min to 390 min.

### 3.3. Meta-Analysis

**Figure 2:**
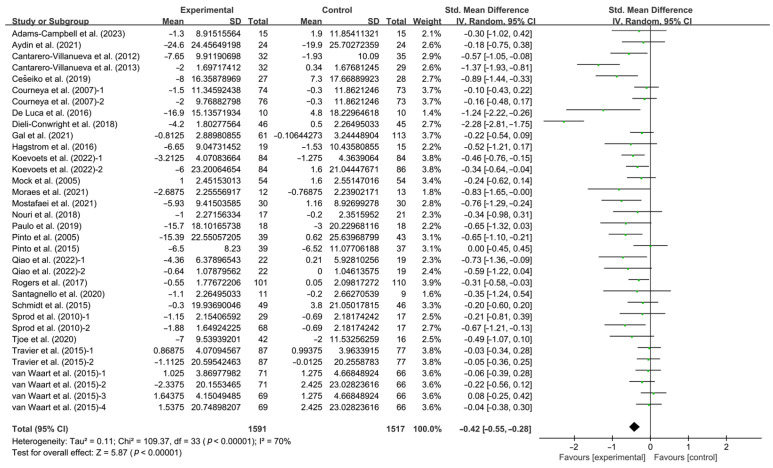
Meta-analysis results of the effects of exercise on CRF in breast cancer patients [32,33,34,35,36,37,38,39,40,41,42,43,44,45,46,47,48,49,50,51,52,53,54,55,56,57]. The size of the shaded squares was proportional to the percentage weight of each study. Diamonds indicated the effect size of each study summarized as SMD.

Exercise was found to have a significant effect on improving CRF in breast cancer patients (SMD, −0.42; 95% CI, −0.55 to −0.28, *p* < 0.0001, I^2^ = 70%, Figure 2). To delve deeper into the variability among the studies and identify potential factors that could be modified to optimize exercise effects, further analyses were conducted, including meta-regression, subgroup, and sensitivity analyses.

### 3.4. Meta-Regression Analysis

Meta-regression analysis was applied to investigate the relationship between CRF improvement, various intervention attributes (intervention duration, session duration, frequency, and weekly time), and participant age. However, no statistically significant associations were observed between CRF enhancement and any of these factors, including intervention duration (*p* = 0.929), frequency (*p* = 0.387), session duration (*p* = 0.364), weekly time (*p* = 0.362), or age (*p* = 0.651), as depicted in Figure S1.

### 3.5. Subgroup Analysis

**Figure 3:**
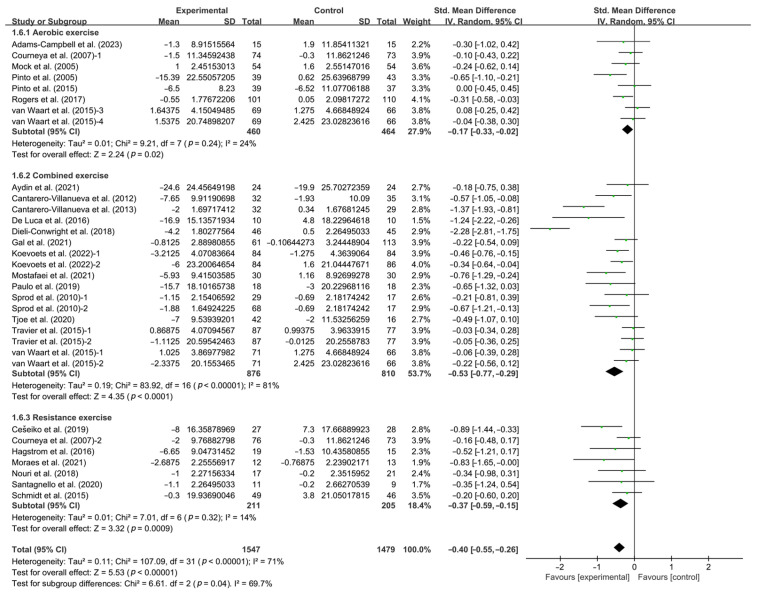
Meta-analysis results of the effects of types of intervention on CRF in breast cancer patients [32,33,34,35,36,37,38,39,40,41,42,43,44,45,46,47,48,49,51,52,53,54,55,56,57]. The size of the shaded squares was proportional to the percentage weight of each study. Diamonds indicated the effect size of each study summarized as SMD.

Stratifying the analysis by types of intervention, aerobic exercise (SMD, −0.17; 95% CI, −0.33 to −0.02, *p* = 0.02, I^2^ = 24%), resistance exercise (SMD, −0.37; 95% CI, −0.59 to −0.15, *p* = 0.0009, I^2^ = 14%), and combined exercise (SMD, −0.53; 95% CI, −0.77 to −0.29, *p* < 0.0001, I^2^ = 81%, Figure 3 and Table 1) significantly improved CRF in breast cancer patients, with combined exercise being the most effective intervention.

**Figure 4:**
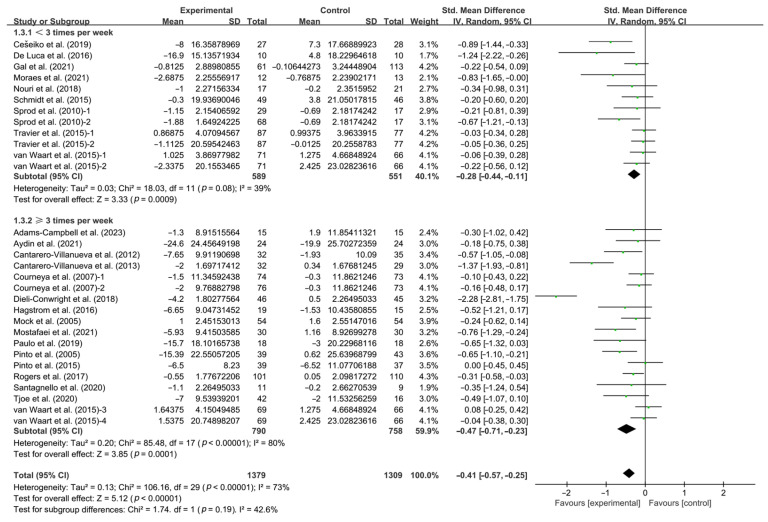
Meta-analysis results of the effects of frequency of intervention on CRF in breast cancer patients [32,33,34,35,36,37,38,39,40,41,43,44,45,46,47,48,49,51,52,53,54,55,56,57]. The size of the shaded squares was proportional to the percentage weight of each study. Diamonds indicated the effect size of each study summarized as SMD.

In addition, when analyzing the subgroups by frequency, interventions conducted for <3 times per week (SMD, −0.28; 95% CI, −0.44 to −0.11, *p* = 0.0009, I^2^ = 39%) and ≥3 times per week (SMD, −0.47; 95% CI, −0.71 to −0.23, *p* = 0.0001, I^2^ = 80%, Figure 4 and Table 1) significantly improved CRF in breast cancer patients, with interventions conducted for ≥3 times per week having a greater effect. 

**Figure 5:**
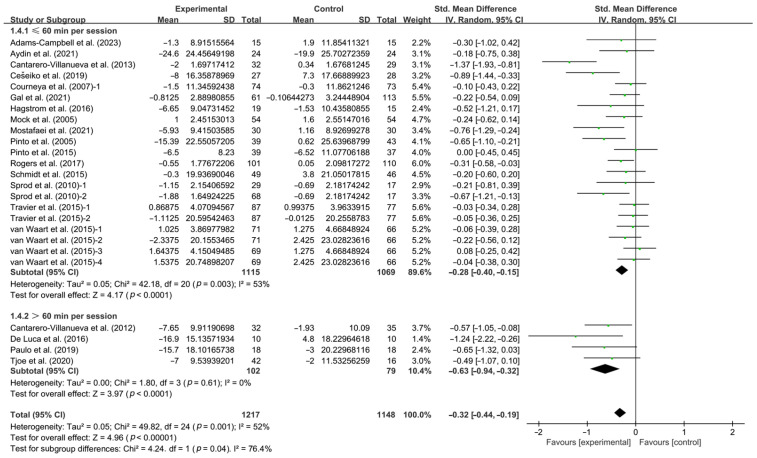
Meta-analysis results of the effects of duration of intervention per session on CRF in breast cancer patients [32,33,34,35,36,37,38,40,41,43,45,47,48,49,51,53,54,55,56,57]. The size of the shaded squares was proportional to the percentage weight of each study. Diamonds indicated the effect size of each study summarized as SMD.

Furthermore, when analyzing the subgroups by session duration, interventions conducted for ≤60 min per session (SMD, −0.28; 95% CI, −0.40 to −0.15, *p* < 0.0001, I^2^ = 53%) and >60 min per session (SMD, −0.63; 95% CI, −0.94 to −0.32, *p* < 0.0001, I^2^ = 0%, Figure 5 and Table 1) significantly improved CRF in breast cancer patients, with interventions conducted for >60 min per session having a greater effect.

**Figure 6:**
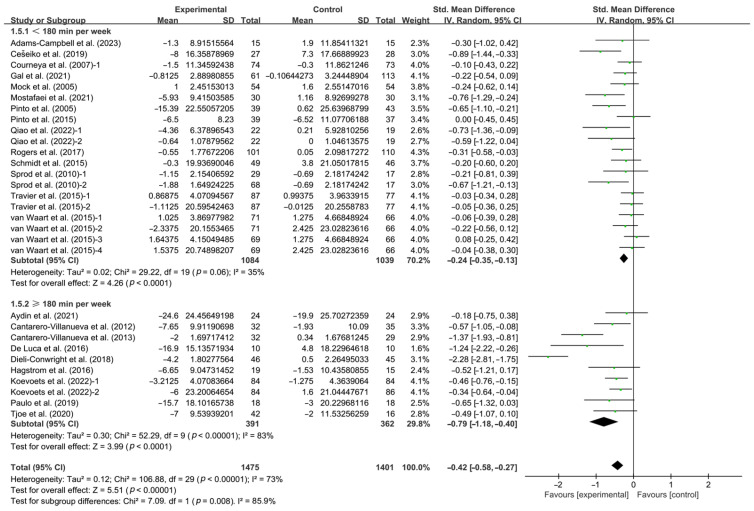
Meta-analysis results of the effects of duration of intervention per week on CRF in breast cancer patients [32,33,34,35,36,37,38,39,40,41,42,43,45,47,48,49,50,51,53,54,55,56,57]. The size of the shaded squares was proportional to the percentage weight of each study. Diamonds indicated the effect size of each study summarized as SMD.

Moreover, when analyzing the subgroups by weekly time, interventions conducted for <180 min per week (SMD, −0.24; 95% CI, −0.35 to −0.13, *p* < 0.0001, I^2^ = 35%) and ≥180 min per week (SMD, −0.79; 95% CI, −1.18 to −0.40, *p* < 0.0001, I^2^ = 83%, Figure 6 and Table 1) significantly improved CRF in breast cancer patients, with interventions conducted for ≥180 min per week having a greater effect.

**Figure 7:**
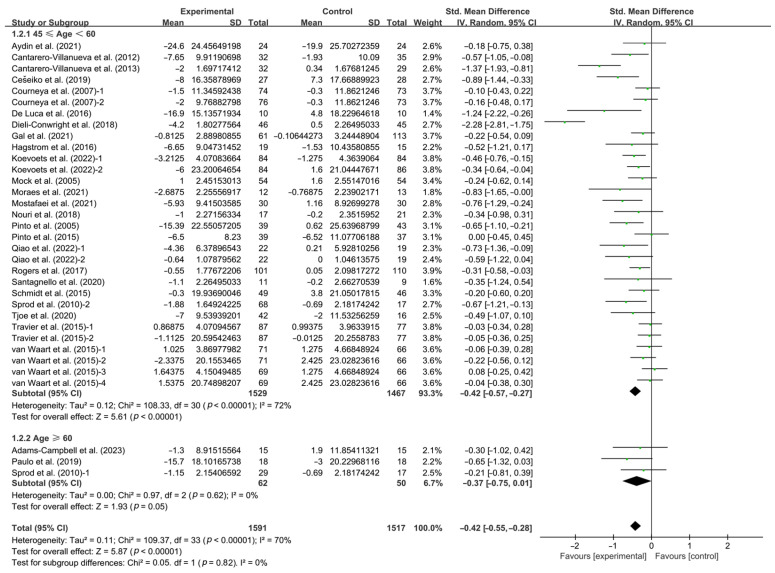
Meta-analysis results of the effects of exercise on CRF in middle-aged and elderly breast cancer patients [32,33,34,35,36,37,38,39,40,41,42,43,44,45,46,47,48,49,50,51,52,53,54,55,56,57]. The size of the shaded squares was proportional to the percentage weight of each study. Diamonds indicated the effect size of each study summarized as SMD.

Finally, when analyzing the subgroups by participant age, exercise significantly improved CRF in middle-aged breast cancer patients (SMD, −0.42; 95% CI, −0.57 to −0.27, *p* < 0.0001, I^2^ = 72%), while exercise had no significant effect on improving CRF in elderly breast cancer patients (SMD, −0.37; 95% CI, −0.75 to 0.01, *p* = 0.05, I^2^ = 0%, Figure 7 and Table 1).

### 3.6. Risk of Bias

The RoB-2 tool was utilized to evaluate the risk of bias in the included studies, considering factors such as selection, performance, detection, attrition, reporting, and other biases. As illustrated in Figure S2, the overall quality of the studies was categorized into three levels: low, moderate, and high. Two studies posed a low risk of bias, twenty-one studies presented a moderate risk, and three studies had a high risk of bias.

### 3.7. Sensitivity Analyses

Sensitivity analyses indicated that the positive impact of exercise on CRF in breast cancer patients remained stable and consistent in both direction and magnitude, regardless of the exclusion of any individual study (Figure S3).

### 3.8. Publication Bias

To further assess the potential for publication bias, a funnel plot analysis was conducted (Figure S4). The observed asymmetry indicates the presence of publication bias.

## 4. Discussion

### 4.1. Main Findings

The present study aimed to assess the effects of exercise on CRF and establish a definitive exercise prescription tailored to the needs of breast cancer patients. A total of 26 studies were included, with the results conclusively demonstrating that exercise significantly improved CRF in breast cancer patients. Further subgroup analyses revealed that combined exercise, undertaken at a frequency of at least three times weekly, each session lasting over 60 min, and accumulating a total of 180 min or more per week, proved to be the most efficacious in improving CRF, particularly in middle-aged breast cancer patients.

### 4.2. Effects of Exercise on CRF in Breast Cancer Patients

This study suggested that exercise holds the potential to improve CRF in breast cancer patients, which is consistent with previous studies [58,59]. There are numerous explanations for the potential mechanisms of how exercise improves CRF in breast cancer patients, and the following are some possible mechanisms that could account for the effects of exercise.

Firstly, research has consistently demonstrated that exercise boosts anti-inflammatory cytokines while reducing pro-inflammatory adipokines [60]. While only a few studies have investigated changes in inflammatory mediators in breast cancer patients following exercise interventions, all of these studies consistently reported a decrease in inflammatory factors like interleukin-6 (IL-6), C-reactive protein (CRP), and tumor necrosis factor-α (TNF-α) post-exercise [61,62]. Since inflammation is a potential cause of CRF, the anti-inflammatory effect of exercise likely contributes to the reduction in fatigue. Additionally, the increase in the number of lymphocytes stimulated by exercise may explain the positive effect of exercise on CRF from an immunological perspective [63].

Secondly, resistance exercise is likely to mitigate muscle function decline, such as muscle atrophy caused by cancer [64]. Studies have demonstrated that resistance exercise improves cytokine responses [65] and enhances generalized muscle strength in cancer patients [66]. However, aerobic exercise enhances energy metabolism processes, where carbohydrates and fats are thoroughly oxidized into water and carbon dioxide within the mitochondria, yielding adenosine triphosphate (ATP) as a stored energy source in cells, thereby enhancing cardiorespiratory fitness among patients [67]. These improvements may enable breast cancer patients to perform daily activities with more ease and at the same intensity as before, thereby reducing the perception of fatigue.

Finally, exercise can also have positive effects on mental health. For instance, achieving daily activity goals can boost self-confidence and self-efficacy, indirectly reducing fatigue. Previous studies have shown that self-efficacy is a mediating factor in reducing fatigue in breast cancer patients [68,69]. Additionally, exercise improves sleep, mood, and cognition, all of which indirectly affects fatigue [70].

However, our results were inconsistent with some previous studies. For instance, Cramp et al. [71] suggested that aerobic exercise significantly improved CRF, while other forms of exercise did not exhibit such an effect. Concurrently, there exists uncertainty in the literature regarding the definitive benefits of exercise on CRF in adult populations [72]. The inconsistency may be attributed to the fact that these studies did not guarantee that all included patients suffered from breast cancer, but that merely a majority did, and the varying characteristics of the interventions focused on in different studies may also have contributed to the inconsistent results.

### 4.3. Subgroup Analysis

Our subgroup analysis showed that aerobic exercise, resistance exercise, and combined exercise significantly improved CRF in breast cancer patients, with combined exercise emerging as the most effective approach, mirroring prior research findings. Steindorf et al. [73] found that resistance exercise significantly reduced CRF after a twelve-week intervention in breast cancer patients. In addition, Yang et al. [74] suggested that moderate-intensity aerobic exercise also achieved improvements, while Mijwel et al. [75] proposed that the combination of resistance and high-intensity interval training (HIIT) was superior to conventional controls in reducing CRF. Furthermore, numerous studies have demonstrated the unique benefits of combined exercise. Milne et al. [25] corroborates our results, revealing that a blend of aerobic and resistance exercises significantly improved health outcomes, including diminished fatigue, within a brief timeframe. However, both aerobic and resistance exercise, as separate modalities, also possess therapeutic effects, we can still justify the use of aerobic and resistance exercise individually in actual treatment, considering the patient’s physical capabilities. For patients without an exercise foundation, we can prioritize aerobic exercise to develop their cardiorespiratory function before gradually incorporating resistance training and ultimately forming a combined exercise regimen. However, there is still no definitive conclusion on how to design the ratio of aerobic and resistance in the combined exercise mode.

In regard to intervention frequency, both less than three and at least three sessions per week significantly improved CRF in breast cancer patients, aligning with prior studies [76,77]. Notably, a frequency of at least three sessions weekly had a more pronounced effect on CRF, potentially due to its role in fostering a regular exercise routine [78]. However, we did not dismiss the potential benefits of interventions conducted less than 3 times per week, which may still be considered in practical applications, taking into account factors such as session duration.

Our subgroup analysis indicated that interventions conducted for up to 60 min per session and over 60 min per session significantly improved CRF in breast cancer patients, which aligns with previous studies. Meneses-Echávez et al. [79] showed that a supervised exercise intervention of 40 min per session significantly reduced CRF. In addition, a meta-analysis conducted by Sweegers et al. [80] on moderators of exercise in cancer patients (two-thirds of whom were breast cancer patients) also showed a significant effect of exercise intervention within 60 min. Furthermore, our results showed that interventions conducted for over 60 min per session had a greater effect on improving CRF, which is consistent with previous studies. For instance, Zhou et al. [81] showed that engaging in exercise for more than 60 min per session significantly alleviate CRF in breast cancer patients. In addition, Danhauer et al. [82] also concluded that even 75 min of low-intensity exercise can improve fatigue symptoms in breast cancer patients. However, it is crucial to acknowledge that excessive exercise durations may not yield additional health benefits and could potentially have adverse effects. Li et al. [83] found that exercise interventions exceeding 60 min did not significantly impact cognitive function in multiple sclerosis patients, suggesting a threshold beyond which further exercise may not be beneficial. Moreover, insufficient exercise durations fail to elicit improvements, while excessive exercise can induce fatigue and compromise brain plasticity. Given that our intervention targeted cancer patients, it is worth noting that exercise tolerance in this population is reduced compared to the healthy population, mainly due to the negative impact of CRF on exercise tolerance [84]. Therefore, we caution against blindly increasing session durations and advocate for potentially more effective improvements through increased frequency.

Nevertheless, our study revealed that merely focusing on frequency and session duration was insufficient to mitigate the influence of other confounding variables. Consequently, we devised a method to calculate the weekly exercise time by combining both these factors. Our findings indicated that interventions totaling at least 180 min per week had a more pronounced effect on improving CRF in breast cancer patients. Therefore, the combination of a frequency of at least three interventions per week and a duration of at least 180 min of intervention per week achieves a better effect, suggesting that the recommended exercise pattern for breast cancer patients should be to appropriately reduce the duration of each exercise intervention to avoid side effects such as muscle injury or functional impairment caused by decreased exercise tolerance, while ensuring an adequate volume of exercise by increasing the frequency of exercise per week to achieve a better therapeutic effect.

In the current study, participants comprised middle-aged and older individuals, and our subgroup analysis showed that exercise significantly improved CRF in middle-aged breast cancer patients. The lack of a significant effect in older patients may be attributed to their reduced exercise tolerance [85], as well as decreased motivation and adherence to exercise compared to middle-aged patients [86]. However, only three trials in this meta-analysis involved participants with a mean age of 60 years or older, necessitating further clinical trials to validate the therapeutic effects of exercise on CRF in older adults.

Exercise interventions during or after other treatments were excluded from this study, which could potentially impact treatment effectiveness based on previous studies. Juvet et al. [77] found that exercise initiated after radiotherapy or chemotherapy was more effective in reducing CRF in breast cancer patients compared to exercise during treatment. Additionally, Hilfiker et al. [59] showed that relaxation exercises were effective during cancer-related treatment, but their effectiveness significantly diminished post-treatment. It has also been proposed that exercise during treatment may have a more favorable and faster effect on mobility compared to post-treatment [87], though further evidence is needed to substantiate this claim.

### 4.4. Strengths and Limitations of This Study

Our strength lies in the thorough analysis of the intervention-related factors, including type of exercise, frequency, session duration, and weekly time. However, this study had certain limitations. The blinding quality of the included studies could not be assured, potentially affecting the robustness of the evidence. In addition, exercise intensity, the supervision of exercise interventions, and rest intervals during exercise were not statistically analyzed. Future studies with larger sample sizes and higher quality may be needed to complement our findings. Finally, there is a high degree of heterogeneity in this study, necessitating careful and appropriate handling of the results.

## 5. Conclusions

Exercise is an effective approach to improving CRF in breast cancer patients. When devising an exercise program, the primary consideration should be the incorporation of combined exercise as the principal intervention. This entails ensuring that participants engage in the program at least three times weekly, with each session lasting for more than 60 min. The ultimate aim is to achieve a total weekly exercise duration of 180 min by progressively increasing the frequency of exercise sessions.

## Figures and Tables

**Table 1:** Results of moderator analysis.

Moderator	SMD (95% CI)	I^2^	*p* Value
**Overall**	−0.42 (−0.55, −0.28)	70%	<0.0001
**Types of intervention**			
Aerobic exercise	−0.17 (−0.33, −0.02)	24%	0.02
Resistance exercise	−0.37 (−0.59, −0.15)	14%	0.0009
Combined exercise	−0.53 (−0.77, −0.29)	81%	<0.0001
**Frequency**			
<3 times per week	−0.28 (−0.44, −0.11)	39%	0.0009
≥3 times per week	−0.47 (−0.71, −0.23)	80%	0.0001
**Session duration**			
≤60 min per session	−0.28 (−0.40, −0.15)	53%	<0.0001
>60 min per session	−0.63 (−0.94, −0.32)	0%	<0.0001
**Weekly time**			
<180 min per week	−0.24 (−0.35, −0.13)	35%	<0.0001
≥180 min per week	−0.79 (−1.18, −0.40)	83%	<0.0001
**Age**			
45 ≤ Age < 60	−0.42 (−0.57, −0.27)	72%	<0.0001
Age ≥60	−0.37 (−0.75, 0.01)	0%	0.05

**Abbreviations:** 95% CI, 95% confidence interval.

## Data Availability

All data generated or analyzed during this study are included in the article/Supplementary Materials.

## Supplementary Materials

The following supporting information can be downloaded at: https://www.mdpi.com/article/10.3390/life14081011/s1, Figure S1: Meta-regression analysis results; Figure S2: Results of Cochrane risk of bias tool; Figure S3: Sensitivity analyses results [32,33,34,35,36,37,38,39,40,41,42,43,44,45,46,47,48,49,50,51,52,53,54,55,56,57]; Figure S4: Funnel plot; Table S1: Characteristics of the studies included in this meta-analysis.
