# Supplementary material for: Effects of Exercise on Cancer-Related Fatigue in Breast Cancer Patients: A Systematic Review and Meta-Analysis of Randomized Controlled Trials
Source: Life (Basel). 2024 Aug 14;14(8):1011. doi: 10.3390/life14081011 (PMC11355832; doi:10.3390/life14081011)
Supplement: Supplementary file 1 [file life-14-01011-s001.zip › life-3084310-supplementary.pdf]

Supplemental material

Effects of Exercise on Cancer-Related Fatigue in Breast Cancer Patients: A Systematic Review and Meta-Analysis of Randomized Controlled Trials

Figure S1 Meta-regression analysis results..2
Figure S2 Results of Cochrane risk of bias tool......................................3
Figure S3 Sensitivity analyses results...4
Figure S4 Funnel plot..5
Table S1 Characteristics of the studies included in this meta-analysis......6

Figure S1 Meta-regression analysis results

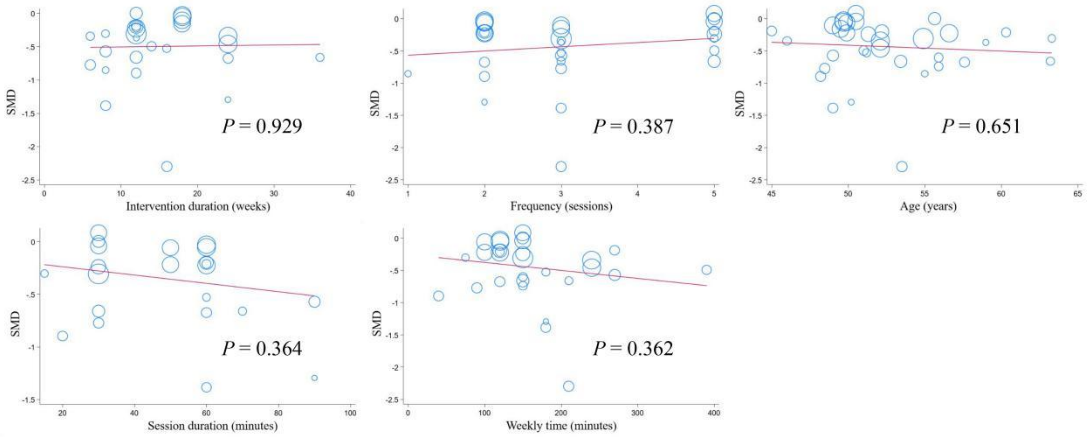

Figure S2 Results of Cochrane risk of bias tool

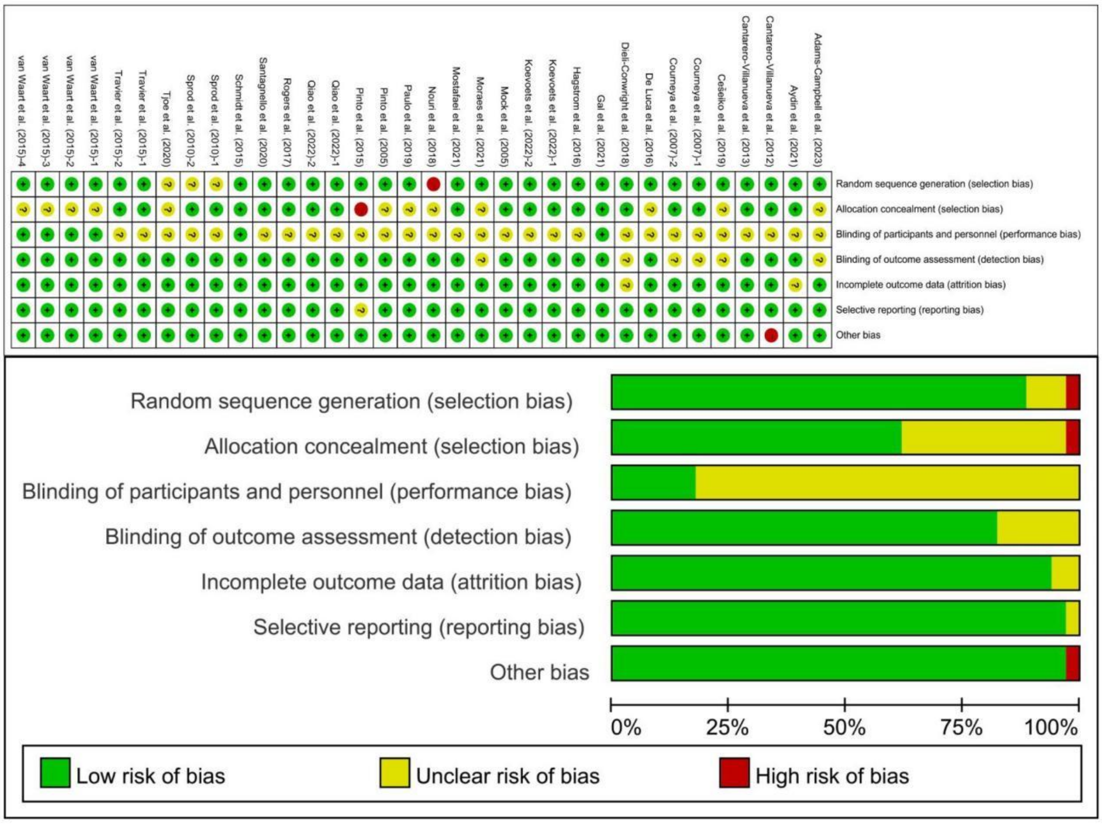

Figure S3 Sensitivity analysis results

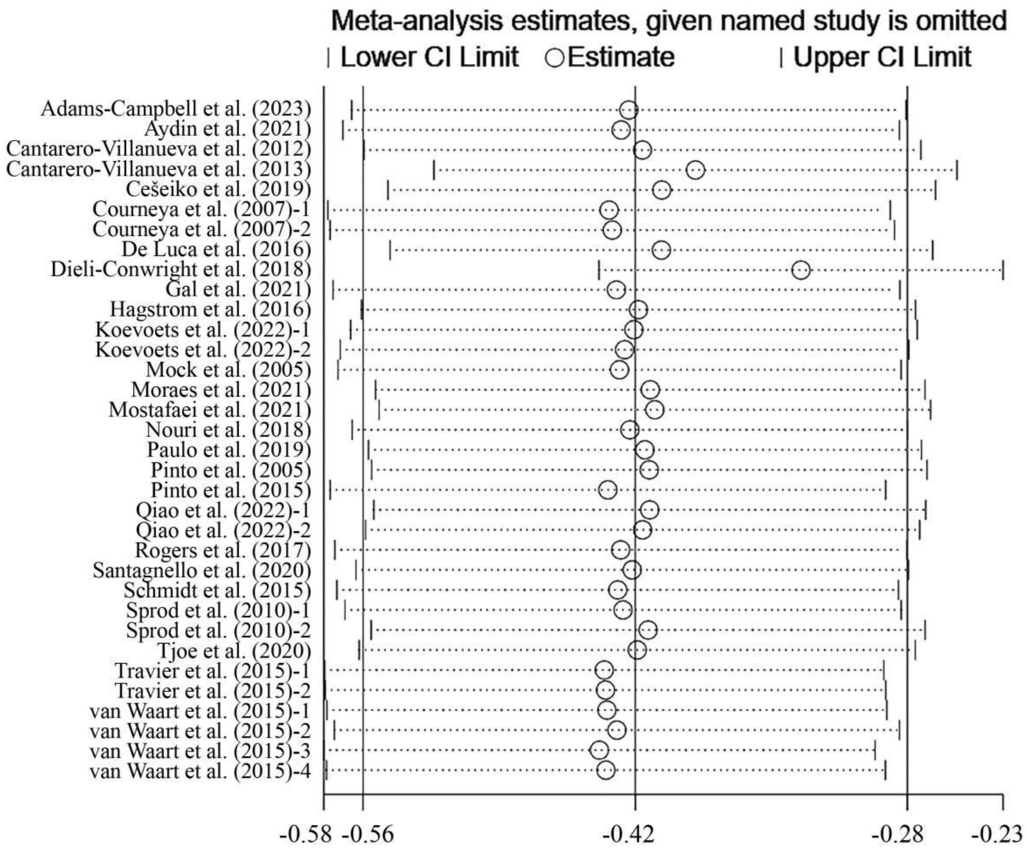

Figure S4 Funnel plot

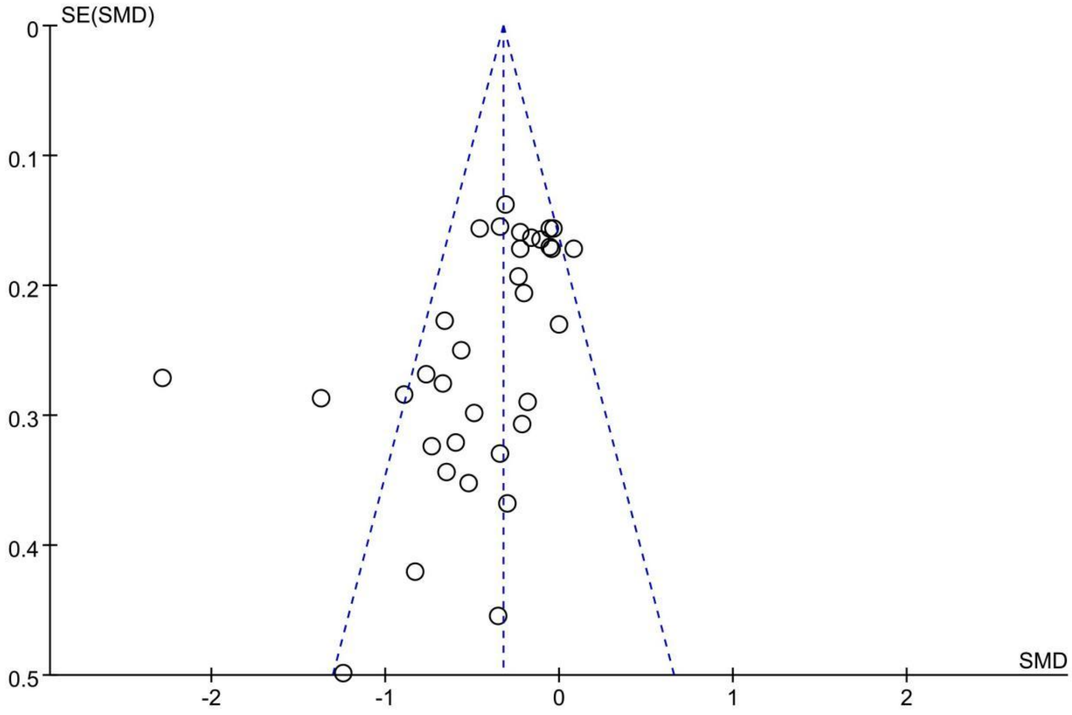

Table S1. Characteristics of the studies included in this meta-analysis

Studies	Sample	Stage	Age (years)	Type	Length (weeks)	Frequency (times/week)	Session (min)	Results on CRF
Adams-Campbell et al. (2023)[32]	IG: 15<br>CG: 15	IG: 0-Ⅱ<br>CG: 0-Ⅲ	IG: 63.3 ± 3.2<br>CG: 64.5 ± 3.2	Aerobic	8	5	15	FACIT-F
Aydin et al. (2021)[33]	IG: 24<br>CG: 24	NC	45.0 ± 2.2	Combined	12	5	110	EORTC QLQ-C30
Cantarero-Villanueva et al. (2012)[34]	IG: 32<br>CG: 35	Ⅰ-ⅢA	IG: 49.0 ± 9.0<br>CG: 48.0 ± 9.0	Combined	8	3	90	POMS
Cantarero-Villanueva et al. (2013)[35]	IG: 32<br>CG: 29	Ⅰ-ⅢA	IG: 49.0 ± 7.0<br>CG: 47.0 ± 8.0	Combined	8	3	60	PFS
Cešeiko et al. (2019)[36]	IG: 27<br>CG: 28	Ⅰ-Ⅲ	IG: 48.2 ± 6.7<br>CG: 49.0 ± 8.0	Resistance	12	2	20	EORTC QLQ-C30
Courneya et al. (2007)[37]	IG1: 74<br>IG2: 76<br>CG: 73	Ⅰ/ⅡA/<br>ⅡB/ⅢA	IG1: 49.0 ± 11.25<br>IG2: 49.5 ±	IG1: Aerobic<br>IG2: Resistance	18	3	IG1: 45 ↑<br>IG2: NC	FACT-An

			12.75<br>CG: 49.0 ± 13					
De Luca et al. (2016)[38]	IG: 10<br>CG: 10	Ⅰ-Ⅲ	IG: 50.2 ± 9.7<br>CG: 46.0 ± 2.8	Combined	24	2	90	FACIT-F
Dieli-Conwright et al. (2018)[39]	IG: 46<br>CG: 45	0-Ⅲ	53.5 ± 10.4	Combined	16	3	130	BFI
Gal et al. (2021)[40]	IG: 61<br>CG: 113	DCIS/Ⅰ-Ⅲ	IG: 56.6 ± 9.8<br>CG: 58.3 ± 9.5	Combined	12	2	60	MFI-20
Hagstrom et al. (2016)[41]	IG: 19<br>CG: 15	Ⅰ-ⅢA	IG: 51.2 ± 8.5<br>CG: 52.7 ± 9.4	Resistance	16	3	60	FACIT-F
Koevoets et al. (2022)[42]	IG: 84<br>CG: 84/86	Ⅰ-Ⅲ/ Unclear	IG: 52.1 ± 8.6<br>CG: 52.5 ± 8.7	Combined	24	NC	NC	MFI; EORTC QLQ-C30
Mock et al. (2005)[43]	IG: 54<br>CG: 54	0-ⅢA	IG: 51.3 ± 8.9<br>CG: 51.6 ± 9.7	Aerobic	6/12-24	5-6	30 ↑	PFS
Moraes et al. (2021)[44]	IG: 12	NC	IG: 55.0 ± 5.8	Resistance	8	1	NC	PFS

	CG: 13		CG: 54.3 ± 5.2					
Mostafaei et al. (2021)[45]	IG: 30<br>CG: 30	0-Ⅲ	IG: 48.46 ± 5.72<br>CG: 49.6 ± 7.48	Combined	6	3	30	FSS
Nouri et al. (2018)[46]	IG: 17<br>CG: 21	0-Ⅲ	IG: 46 ± 5.8<br>CG: 46 ± 7.2	Resistance	6	⩽ 3	NC	PFS
Paulo et al. (2019)[47]	IG: 18<br>CG: 18	Ⅰ-Ⅲ	IG: 63.2 ± 7.1<br>CG: 66.6 ± 9.6	Combined	36	3	70	EORTC QLQ-C30
Pinto et al. (2005)[48]	IG: 39<br>CG: 43	0-Ⅱ	IG: 53.42 ± 9.08<br>CG: 52.86 ± 10.38	Aerobic	12	⩾ 5	30	10-cm linear analog scale
Pinto et al. (2015)[49]	IG: 39<br>CG: 37	0-Ⅲ	IG: 55.64 ± 8.59<br>CG: 55.59 ± 10.59	Aerobic	12	⩾ 5	⩾ 30	FACIT-F
Qiao et al. (2022)[50]	IG: 22	NC	IG: 55.9 ± 8.2	NC	6-14	NC	NC	PFS²;

	CG: 19		CG: 53.8 ± 11.4					FACT-ES
Rogers et al. (2017)[51]	IG: 101<br>CG: 110	DCIS/ Ⅰ-Ⅲ	IG: 54.9 ± 9.3<br>CG: 53.9 ± 7.7	Aerobic	12	⩾ 3	30-50	FSI
Santagnello et al. (2020)[52]	IG: 11<br>CG: 9	Ⅰ-Ⅲ	IG: 59.0 ± 9.2<br>CG: 52.1 ± 10.1	Resistance	12	3	NC	BFI
Schmidt et al. (2015)[53]	IG: 49<br>CG: 46	Ⅰ-Ⅳ	IG: 52.2 ± 9.9<br>CG: 53.3 ± 10.2	Resistance	12	2	60	FAQ
Sprod et al. (2010)[54]	IG1: 29<br>IG2: 68<br>CG: 17	NC	IG1: 60.3 ± 2.4<br>IG2: 57.6 ± 1.2<br>CG: 61.2 ± 4.0	Combined	IG1: 12<br>IG2: 24	2-3	60	PFS
Tjoe et al. (2020)[55]	IG: 42<br>CG: 16	IG: 0-Ⅲ<br>CG: Ⅰ-Ⅳ	IG: 51.0 ± 7.0<br>CG: 56.0 ± 10.0	Combined	14	5-6	90	FACIT-F
Travier et al. (2015)[56]	IG: 87<br>CG: 77	M0	IG: 49.7 ± 8.2<br>CG: 49.5 ± 7.9	Combined	18	2	60	MFI; FQL

van Waart et al. (2015)[57]	IG1: 71<br>IG2: 69<br>CG: 66	Ⅰ-Ⅲ	IG1: 49.9 ± 8.4<br>IG2: 50.5 ± 10.1<br>CG: 51.6 ± 8.8	IG1: Combined<br>IG2: Aerobic	NC	IG1: 2<br>IG2: 5	IG1: 50<br>IG2: ⩾ 30	MFI; FQL

Abbreviation: IG, intervention groups; CG, control groups; NC, not clear; DCIS, ductal carcinoma in situ; M0, no distant metastasis; ↑, gradually increase intervention time each week until target time; FACIT-F, the Functional Assessment of Chronic Illness Therapy-Fatigue; EORTC QLQ-C30, the European Organization for Research and Treatment of Cancer Core Quality of Life Questionnaire-C30; POMS, the Profile of Mood States; PFS, the Piper Fatigue Scale/the revised Piper Fatigue Scale; FACT-An, the Functional Assessment of Cancer Therapy-Anemia scale; BFI, the Brief Fatigue Inventory; MFI/MFI-20, the Multidimensional Fatigue Inventory; FSS, the Fatigue severity scale; PFS2, the Pittsburgh Fatigability Scale; FACT-ES, the Functional Assessment of Cancer Therapy-Endocrine Symptoms questionnaire; FSI, the Fatigue Symptom Inventory; FAQ, the Fatigue Assessment Questionnaire; FQL, the Fatigue Quality List.
